# Traditional Chinese Medicines for Alzheimer's Disease: Current Knowledge, Clinical Applications, and Future Directions

**DOI:** 10.2174/0115680266347052250407110353

**Published:** 2025-05-12

**Authors:** Yu Deng, Chaojun Chen, Hongtao Li, Tianle Wang, Xu Zhang, Xueyang Wang, Guangtao Pan

**Affiliations:** 1 Guangzhou University of Chinese Medicine, Guangzhou 510006, Guangdong, China;; 2 Department of Neurology, Guangzhou Hospital of Integrated Traditional and West Medicine Affiliated to Guangzhou University of Chinese Medicine, Guangzhou 510800, Guangdong, China;; 3 First Affiliated Hospital of Guangzhou University of Chinese Medicine, Guangzhou 510405, Guangdong, China;; 4 Yancheng TCM Hospital Affiliated to Nanjing University of Chinese Medicine, Yancheng, 224000, Jiangsu, China;; 5 Faculty of Chinese Medicine, Macau University of Science and Technology, Avenida Wai Long, Taipa, Macau, China

**Keywords:** Alzheimer's disease, Traditional chinese medicine, Traditional chinese medicine compound recipes, Research progress

## Abstract

Alzheimer's disease (AD) is a prevalent neurodegenerative disorder that poses a significant challenge to the health of the global aging population. Despite extensive research, the complex mechanisms underlying AD pathogenesis remain largely elusive. In recent years, a growing number of clinical studies have demonstrated the preventive and therapeutic potential of Traditional Chinese Medicine (TCM) against AD through multiple pathways, targets, and compounds. In this study, we conducted a review of the literature published over the past 20 years through international and domestic databases, including PubMed, Medline, Cochrane Library, CNKI, SinoMed, Wanfang, and VIP Journal Integration Platform. This review systematically evaluates current research advancements regarding single-herb preparations, bioactive constituents, and compound formulations in Traditional Chinese Medicine (TCM), with focused analysis on three therapeutic categories: tonifying herbs, blood-activating and stasis-eliminating agents, as well as orifice-opening, phlegm-resolving, and mind-stabilizing medicinal substances. Furthermore, this review discusses the potential mechanisms underpinning the anti-AD effects of TCMs. By integrating these insights, this review aims to establish a theoretical foundation for the application of TCMs in AD treatment and provide a reference for future pharmacological studies and the development of health-related products.

## 1 INTRODUCTION

Alzheimer's disease (AD) is a primary central nervous system disorder characterized by cognitive dysfunction. This neurodegenerative condition predominantly affects individuals in their mid- to late-life stages, clinically manifesting as progressive memory decline, language impairment, spatial disorientation, emotional instability, and behavioral disturbances. These manifestations frequently lead to a loss of self-care ability in the affected individuals and, in severe cases, pose a life-threatening risk [1]. Recent years have witnessed a growing body of studies regarding the pathogenic mechanisms underpinning AD, which are critical to understanding and addressing this complex disease. For more than 3,000 years, Traditional Chinese Medicine (TCM) has been widely used in China and other Asian countries for the prevention and treatment of neurodegenerative diseases [2]. TCM therapies employ multi-targeted and multi-pathway approaches to address the complex and multifactorial nature of neurodegenerative disorders. In recent years, researchers have demonstrated that numerous herbal medicines and their isolated bioactive components have promising therapeutic efficacy against AD, often accompanied by a lower incidence of adverse effects compared to conventional treatments. Extracts derived from *Ginkgo biloba*, such as EGB761, have been shown to improve cognitive function, reduce neuropsychiatric symptoms, and enhance functional capacity in AD patients [3].

Furthermore, monomeric compounds extracted from various herbal sources, such as baicalein [4], tanshinone [5], and stilbenoids [6], have also highlighted promising therapeutic effects against AD. Accumulating studies have found that blood-activating and blockage-removing TCMs [7] and qi-benefiting and phlegm-removing TCMs [8] exert beneficial effects in the treatment of AD. Despite accumulating evidence supporting the therapeutic effects of TCMs for AD, no comprehensive study has yet categorized representative herbal medicines and traditional formulations based on their specific therapeutic actions against AD pathology. Therefore, this comprehensive review systematically evaluates the pathophysiological mechanisms underlying Alzheimer's disease (AD) and critically appraises the therapeutic potential of various Traditional Chinese Medicine (TCM) modalities, including bioactive constituents, phytochemical derivatives, and classical herbal formulations, through a therapeutic efficacy-based classification framework. The analysis aims to advance research paradigms by identifying novel therapeutic targets and elucidating the molecular mechanisms through which TCM interventions exert neuroprotective effects in AD management.

### 1.1 Overview of the pathogenesis of AD

The development of AD is a complex process primarily characterized by the accumulation of amyloid β-protein (Aβ) in the brain. This accumulation triggers sustained and excessive activation of microglial cells and stimulates the secretion of various inflammatory factors, thereby initiating a neuroinflammatory response [9]. Concurrently, the tau protein, essential in maintaining neuronal structural integrity and function, undergoes hyperphosphorylation in response to these inflammatory reactions. Normally, tau, a microtubule-binding protein, plays crucial roles in stabilizing microtubule structures, maintaining cellular scaffolding, facilitating intracellular transport, and mediating signal transduction [10]. However, in the context of AD, the hyperphosphorylation of tau reduces its microtubule-binding capacity and increases its propensity to aggregate, thereby resulting in the formation of Neurofibrillary Tangles (NFTs). This cascade of events contributes to axonal damage and neuron apoptosis [11]. Moreover, the inflammation responses in AD are also associated with reduced neurotransmitter activities.

Notably, the loss of cholinergic neurons in the basal nucleus, accompanied by reductions in Acetylcholine (ACh) levels and alterations in Choline Acetyltransferase (ChAT) and Acetylcholinesterase (AChE), is critically involved in learning and memory impairments in the context of AD [12]. In addition, these pathological changes, including inflammatory responses, adversely affect the synaptic plasticity of neurons. Synaptic plasticity, a key indicator of neural adaptability, and synaptic damage or loss are prominent early pathological alterations in AD. Improving synaptic morphology and promoting synaptic transmission plasticity are promising strategies for mitigating cognitive decline in AD patients [13, 14]. Beyond these mechanisms, several other factors are implicated in the development of AD. Excitotoxicity, primarily caused by Excess Excitatory Amino Acids (EAAs) like glutamate, results in neuronal damage [15]. This process is accompanied by oxidative stress, where an overload of free radicals damages cells, playing a significant role in neuronal degeneration in AD [16]. Furthermore, calcium dysregulation in neurons disrupts cellular homeostasis and causes neuronal cell death [17]. Hormonal changes, especially a decrease in estrogen levels, have been linked to a heightened risk of AD, suggesting neuroprotective properties of estrogen [18]. Mitochondrial dysfunction, characterized by reduced energy production and increased oxidative stress, exacerbates neuronal damage in AD [19]. Insulin signaling abnormalities affect brain function and are implicated in cognitive decline [20]. Emerging evidence also points to the role of gut microbiome disturbances in AD. Changes in the gut-brain axis may influence systemic inflammation and metabolic functions to impact the progression of AD [21]. In summary, the pathogenesis of AD is a complex interplay of Aβ accumulation, microglial activation, tau hyperphosphorylation, neurotransmitter dysfunction, synaptic impairment, oxidative stress, excitotoxicity, calcium imbalance, hormonal changes, mitochondrial and insulin signaling abnormalities, and gut microbiome alterations. Together, these factors contribute to the oxidative stress injuries and the progressive neurodegeneration in AD.

## 2 TCM-BASED ANALYSIS FOR THE PATHOGENESIS OF AD

In the context of TCM, AD is commonly referred to as “dementia,” known as “Chidai” in Chinese. TCM displays a profoundly established and comprehensive understanding of this condition. In the context of TCM, dementia is viewed as a brain-centered disorder closely involved with the functions of the five internal organs: heart, liver, spleen, lungs, and kidneys. The heart is considered the central organ overseeing mental activities and is described as the master of the five viscera and six bowels. Dysfunction in the heart’s function impairs its mastery over the body's other organs, which may elevate the risk of dementia. The kidneys, viewed as the foundation of a person's innate nature, are responsible for storing essence and producing marrow for the brain, directly linking their vitality to the development of AD. The spleen plays an essential role in transporting and transforming nutrients, forming the basis for the production of qi and blood. When the spleen is weakened, it fails to adequately nourish the brain. The liver, as the primary regulator of qi and emotional states, also contributes significantly to brain health. Any disturbances in liver function, especially those resulting in qi stagnation, are recognized as key factors in the development of AD.

Furthermore, the lungs, which regulate respiration, qi distribution, and fluid management, are crucial organs that, when dysfunctional, can also contribute to dementia. According to TCM principles, the primary causes of dementia are deficiencies in the “marrow sea,” obstruction of orifices due to phlegm and blood stasis, and dysfunction in vital essence. Other contributing factors include imbalances, such as heart and liver fire, dual deficiency of the spleen and kidneys, yin deficiency in the liver and kidneys, as well as the stagnation of qi and blood stasis.

The aging process naturally leads to a gradual decline in the functions of various organs in older individuals. This decline is particularly evident in the liver and kidneys, where a progressive insufficiency is often observed. Subsequently, the healthy qi or vital energy of the body diminishes, making it more vulnerable to external toxins and internal pathogenic factors. This vulnerability facilitates the accumulation of phlegm, turbidity, and stagnant blood, which then ascend to affect the orifices of the brain, ultimately leading to the development of dementia [22]. Considering the pathogenesis of AD from both TCM and Western medicine perspectives, there are notable similarities in their understanding of the pathological characteristics of this disease. The reduced synaptic plasticity and decreased neurotransmitter activity of AD in Western medicine can be interpreted as a gradual decline in healthy (anti-pathogenic) qi and a progressive weakening of organ functions, as described in TCM. Furthermore, the inflammatory responses in AD can also be understood in TCM as a battle between the anti-pathogenic qi and various pathogenic factors. This integrative perspective enriches the understanding of the occurrence and progression of AD and offers valuable insights into potential treatment strategies.

Historical records indicate that ancient Chinese medical practitioners recognized the potential of natural plants to alleviate memory impairment and cognitive dysfunction. Based on these early observations, successive generations of healers refined and expanded the clinical use of TCMs for the treatment of cognitive and neurodevelopmental disorders. With the advancement of modern medical science, researchers have extracted active ingredients from TCMs, leading to the exploration of their therapeutic potential in AD. Clinical studies have subsequently confirmed that many TCMs and herbal formulas exert beneficial effects in the management of AD [23]. Currently, the use of TCMs remains predominantly centered in East Asian countries; however, with the expansion of global economic and cultural exchanges, interest in the adoption of TCM therapies has also emerged in several European and American countries. In the context of modern medicine, the pharmacological treatment of AD primarily relies on EAA receptor antagonists, cholinesterase inhibitors, ergot alkaloids, and antioxidants, such as rivastigmine, donepezil, huperzine A, and memantine.

Nevertheless, these medications have shown limitations in their capacity to delay or halt the progression of AD [24]. In contrast, TCM employs a different therapeutic strategy for AD, as identified by previous researchers [25]. TCM treatments for AD primarily focus on categories, such as nourishing tonic TCMs, blood circulation-promoting and stasis-resolving TCMs, and TCMs for opening orifices, which resolve phlegm and calm the mind. This article provides a comprehensive review of TCMs in the following categories: active ingredients, TCM compound recipes (TCMCRs), and patent medicines, with the aim of contributing additional insights to the research on TCMs for AD.

## 3 NOURISHING TONIC TCMS

### 3.1 TCMs and their Active Ingredients

#### 3.1.1 Astragalus Radix (Huang Qi)

*Astragalus Radix* (huang qi) is the dried root of *Astragalus membranaceus* (Fisch.) Bge. var. *mongholicus* (Bge.) Hsiao or *Astragalus membranaceus* (Fisch.) Bge. Modern research has identified *Astragalus Radix* as a rich source of trace elements that enhance immune function and exhibit antibacterial properties. In addition to its mineral content, *Astragalus Radix* contains a variety of active ingredients, including polysaccharides, saponins, and isoflavones. Recent experimental studies [26] further revealed that *Astragalus* polysaccharides (APSs) notably facilitated the activities of Superoxide Dismutase (SOD) and Catalase (CAT) in Aβ injection-induced AD rat models, while reducing Malondialdehyde (MDA) levels. These findings suggested that APSs enhanced the endogenous oxygen-free radical scavenging system in AD rat models, thereby repressing lipid peroxidation and ameliorating oxidative stress. Moreover, APSs conferred neuroprotective properties by curtailing Aβ-induced neurotoxicity. Another compound, Astragaloside IV (AS-IV), showed potential in mitigating cognitive dysfunction and suppressing Aβ deposition in the brains of Amyloid Precursor Protein (APP)/PS1 transgenic mouse models. AS-IV could attenuate Aβ_1-42_-induced neurotoxicity and foster the viability of SKN-SH cells, further affirming its neuroprotective effects [27]. Formononetin (FMN), an isoflavone phytoestrogen extracted from Astragalus, is recognized for diverse pharmacological activities, such as anti-cancer, anti-diabetic, neuroprotective, and cardioprotective properties. In a study by Zhang Yingbo and Fei Hongxin [28], an AD mouse model was established using bilateral hippocampal injections of Aβ_1-42_ and intraperitoneal injections of D-galactose (D-gal). The study, which involved continuous treatment with FMN for 35 days, assessed brain water content and Evans Blue content in the mice. The results of immunohistochemistry, real-time quantitative polymerase chain reaction, and Western blot demonstrated that FMN treatment markedly reduced the permeability of the Blood-brain Barrier (BBB) and increased hippocampal zonula occludens-1 (ZO-1) expression in AD mouse models. Both high- and medium-dose FMN treatment disrupted BBB permeability and augmented hippocampal ZO-1 expression. These findings indicate that FMN can effectively diminish BBB permeability in AD mouse models by upregulating hippocampal ZO-1 expression.

#### 3.1.2 Ginseng Radix et Rhizoma (Ren Shen)

*Ginseng Radix et Rhizoma*, derived from the dried roots of the *Panax ginseng* C. A. Mey., is recognized for its anti-aging properties and capacity to modulate nervous system function. Its primary active ingredients are saponins, polysaccharides, and proteins. Modern pharmacological research indicates that its anti-AD effects are predominantly associated with its ability to reduce oxidative stress, diminish inflammation, modulate neurotransmitters, and prevent neuronal apoptosis in the brain. The research by Yan *et al*. [29] identified that a water-soluble ginseng extract (62.5 μg/mL) can render anti-AD effects by repressing AChE activity and protecting human neuroblastoma cells (SH-SY5Y). Furthermore, Zhao *et al*. [30] investigated the impact of total ginsenosides on the memory of aging male C57BL/6J mice. Their study revealed that continuous intragastric administration of ginsenosides ameliorated cognitive impairments in aging mice. This effect was associated with enhanced activities of SOD and glutathione peroxidase (GSH-Px), suggesting neuroprotective effects of ginsenosides through oxidative stress reduction. The study by Liu Shunan [31] on ginseng polysaccharides (GPs) in AD rat models induced by Aβ_25-35_ showed significant improvements in learning and memory abilities, reduced neuronal loss in the hippocampal CA1 region, and restricted neuronal apoptosis. These effects were speculated to be associated with decreased levels of phosphorylated (p-) tau protein and Nuclear Factor κB (NF-κB) p65 and increased MAP2 expression in the hippocampal CA1 region. Zhang *et al*. (2021) [32] established wild-type and APP/PS1 AD mouse models and observed the behavioral changes through open field tests and the Morris Water Maze (MWM). Their findings uncovered that Rg1 treatment for 12 weeks significantly alleviated cognitive deficits and neuronal damage in APP/PS1 mice. Mechanistically, Rg1 reduced p-tau levels, APP expression, Aβ accumulation, ROS generation, and NOX2 expression in the hippocampus and cortex. Yanling Shan *et al*. [33] assessed the effects of different concentrations of ginseng protopanaxdiol (PPD) on two nematode models (CL4176 and BR5270). They observed that PPD delayed paralysis in CL4176 nematodes, extended the lifespan of BR5270, and improved oscillation number and associative memory, indicating its efficacy in mitigating Aβ toxicity and tau protein phosphorylation, improving learning and memory ability, and providing neuroprotection.

#### 3.1.3 Atractylodis Rhizoma (Cang Zhu)

*Atractylodis Rhizoma* (Cang Zhu), derived from the dried roots of *Atractylodes lancea* (Thunb.) DC. or *Atractylodes chinensis* (*DC.*) Koidz., contains various active ingredients, such as polysaccharides, lactones, and multiple amino acids. AM and its extracts exhibit a wide range of pharmacological properties, including anti-microbial, anti-inflammatory, anti-cancer, antioxidant, anti-osteoporosis, and neuroprotective effects [34]. Previous research [35] indicated that AM polysaccharides (AMPs) notably augmented neuronal growth and restricted mitochondrial damage and apoptosis in neurons under hypoxic conditions by elevating Bcl-2 levels and the Bcl-2/Bax ratio. Biatractylolide, as one of the key active components in AM volatile oil, has been found to protect neurons against Aβ_25-35_-induced injury. In the experiments of Feng *et al*. [36], biatractylolide was administered to an AD rat model induced by Aβ_1-40_. Their MWM tests and AChE activity measurement results indicated significant improvements in learning and memory in the biatractylolide-treated rats, evidenced by reduced time spent in the MWM, fewer errors, and decreased AChE activity. These findings suggested that biatractylolide might ameliorate cognitive impairments in rats with dementia-like behaviors by increasing ACh levels in brain regions associated with learning and memory. AM targeted the muscarinic acetylcholine receptor subtype M2 [37], which could lower neuronal reactive oxygen species (ROS) accumulation, diminish oxidative stress, and protect against cellular damage. This receptor was considered a crucial target for the therapeutic action of AM in AD. Furthermore, another animal experimental study [38] demonstrated that water extracts of AM could enhance the memory of D-gal-induced aging mice. Learning and memory are closely associated with changes in the morphology and function of neurons and synapses in brain regions, such as the hippocampus. Hence, these findings imply that AM may boost learning and memory abilities by fostering the expression of synaptic proteins, such as Syn, PKC, and CREB.

#### 3.1.4 Rehmanniae Radix (Di Huang)

*Rehmanniae Radix* derived from the tuberous roots of *Rehmannia glutinosa* Libosch., contains various constituents, including catalpol, rehmannioside, rehmannin, and amino acids. Recognized for its ability to bolster the human immune system, processed *Rehmanniae Radix* (PRR) demonstrates antioxidative properties, improves cognitive functions, and delays the aging process [39]. In the context of AD, PRR has been documented to mitigate oxidative stress, which in turn impeded neuronal apoptosis and improved learning and memory abilities [40]. Prior evidence [41] has underscored that water extracts of PRR reduced MDA levels in the brains of D-gal-induced aging rats, enhanced SOD activity, and upregulated Erythropoietin (EPO) expression, thereby exhibiting antioxidant and stress-reducing effects. The study by Miao *et al*. [42] delineated that PRR polysaccharides facilitated the activities of blood SOD, CAT, and GSH-Px in the D-gal-induced subacute aging mouse model. This improvement was accompanied by reduced peroxidized lipids in plasma, brain homogenates, and liver homogenates, demonstrating substantial antioxidative capabilities.

Additionally, Cui *et al*. [43] revealed that PRR stimulated the production of endogenous neurotrophic factors in the damaged hypothalamic arcuate nucleus of rats, thereby ameliorating neuronal injuries. Hu *et al*. [44] used the SH-SY5Y cell model to assess the impact of PRR on Aβ aggregation. The data from these experiments demonstrated that PRR rendered neuroprotective effects for neurons against Aβ-induced injury. PRR was found to reduce the production and deposition of Aβ in the brain in the treatment of AD.

#### 3.1.5 Summary of several crucial TCMs

Table **1** summarizes several key TCMs, illustrating their active ingredients and mechanisms.

### 3.2 Traditional Chinese Medicine Compound Recipes (TCMCRs)

#### 3.2.1 Bushen-Yizhi Formula (BSYZ)

BSYZ is composed of 11 medicinal ingredients, including Lycium barbarum, Codonopsis pilosula, fruits of Cornus officinalis, AM, PRR, Alpiniae Oxyphyllae Fructus, Curcumae Radix, Angelica sinensis, Acorus tatarinowii, Polygala tenuifolia, and Ligusticum wallichii. BSYZ has demonstrated notable clinical efficacy in the treatment of mild cognitive impairment in older individuals. Its effects might be achieved through nourishing the kidney, invigorating the brain, promoting blood circulation, replenishing qi, awakening the mind, and opening orifices [60]. Hou *et al*. [61] established a rat model of AD using D-gal and Ibotenic Acid (IBO). The experiments revealed that continuous administration of BSYZ at low, medium, and high doses (1.46, 2.92, and 5.84 g/ [kg·d]) for 4 weeks improved the learning and memory of AD rat models. The proposed mechanisms underlying the anti-AD effects of BSYZ include modulation of the Nerve Growth Factor (NGF) signaling pathway and the central cholinergic system. Moreover, Cai *et al*. [62] conducted a systematic pharmacological study on APP/PS1 mice, an AD model, and observed that BSYZ could mitigate AD by modulating Aβ metabolism and impeding neuronal apoptosis. Additional studies suggested that BSYZ might alleviate AD symptoms by reducing oxidative stress and suppressing brain inflammation [63, 64]. Hence, the therapeutic impact of BSYZ on cognitive impairment in APP/PS1 mice may be attributed to its regulation of Aβ metabolism and its capacity to slow down neuronal apoptosis [58].

#### 3.2.2 Dihuang Yinzi (DHYZ)

DHYZ is composed of 15 herbs, including Cistanche deserticola, Morinda officinalis, PRR, Cornus officinalis, monkshood (lateral root of Aconitum carmichaeli), Cinnamomum cassia, tuberous root of Ophiopogon japonicus, Dendrobium nobile, Schisandra chinensis, Polygala tenuifolia, Acorus tatarinowii, Poria cocos, Mentha haplocalyx, fresh rhizoma of Zingiber officinale, and Ziziphus jujuba fructus. DHYZ is known for its kidney-nourishing, essence-replenishing, phlegm-resolving, and orifice-opening effects. Previous clinical studies indicated that DHYZ noticeably enhanced cognitive function in AD patients with minimal adverse effects [65]. The study by Zhang and Wang *et al*. (2018) [66] demonstrated that AD patients treated with DHYZ compound granules (one dose daily for 3 months) exhibited notable increases in serum levels of Notch1 signaling protein and a disintegrin and metalloproteinase 10 (ADAM10), as detected by immunoblotting. Concurrently, the levels of β-site amyloid precursor protein cleaving enzyme 1 (BACE1) were decreased, as measured by enzyme-linked immunosorbent assay. These findings suggest that the potential anti-AD mechanism of BHYZ may involve enhancing Notch1 signaling to promote neuroregeneration, upregulating ADAM10, and lowering BACE1 levels, thereby facilitating APP degradation and rendering neuroprotective effects. In addition, DHYZ has been observed to improve cognitive function and energy metabolism in APP/PS1 mice with AD-like behaviors by protecting mitochondria from pathological damage [67].

#### 3.2.3 Liuwei Dihuang Decoction (LWDHD)

LWDHD is composed of six herbs, including *Poria cocos*, *Rhizoma Dioscoreae*, *Cornus officinalis*, *Cortex Moutan*, *Alismatis Rhizoma*, and PRR. It has been extensively recognized for its capabilities in nourishing yin and tonifying the kidneys and its efficacy in alleviating symptoms related to kidney yin deficiency. Furthermore, LWDHD demonstrates blood glucose-lowering properties and offers neuroprotective benefits. It can enhance the immune system, improve free radical metabolism, and support brain function, conferring benefits for patients with AD. In a study by Li Mengning [68] on the anti-AD mechanism of LWDHD, 10-month-old APP/PS1 double-transgenic male mice were used as AD models. The study unveiled that continuous intragastric administration of LWDHD at a dosage of 10 g/(kg·d) for 117 days increased the number of Nissl bodies in the hippocampal CA3 region in mice with AD-like phenotypes. It also led to a reduction in glial cell activation and an increase in the number of neurons in the brains with AD-like phenotypes, indicating a neuroprotective effect of LWDHD. Several studies also highlighted the role of Rehmannia glycosides, a component of LWDHD, in improving cognitive impairment and treating AD by modulating the immune system and the gut microbiota [69, 70].

#### 3.2.4 Collection of Several Crucial TCMCRs

Several key TCMCRs are presented in Table **2**, offering a detailed overview of their composition, efficacy, and mechanisms.

## 4 TCMS FOR PROMOTING BLOOD CIRCULATION AND RESOLVING STASIS

### 4.1 TCMs and their Active Ingredients

#### 4.1.1 Salvia miltiorrhizae Radix et Rhizoma (Dan Shen)

*Salvia miltiorrhizae Radix et Rhizoma*, derived from the dried roots of *Salvia miltiorrhiza* Bge., is a valued herb in TCM. Modern research has highlighted the primary bioactive ingredients of this herb, which include water-soluble phenolic acids (phenolic acid compounds) and lipid-soluble quinone compounds (tanshinones), as well as components, such as danshensu and cryptotanshinone. These compounds contribute to several therapeutic benefits, including anti-atherosclerotic, anti-inflammatory, and antioxidative stress properties. Moreover, they play a significant role in improving blood circulation and reducing tissue damage caused by ischemia-reperfusion injury [75]. Zhang *et al*. (2019) [76] established an AD cell model by inducing injury in Human Brain Microvascular Endothelial Cells (hBMECs) with Aβ_1-42_ oligomers. The results showed that the extract of *Salvia miltiorrhiza* enhanced the cellular activity in this AD model, and it had a protective effect on the cells of the AD model. Another investigation by Zhang *et al*. (2016) [77] into the mechanisms of total polysaccharides of *Salvia miltiorrhiza* in an APP mouse model revealed that oral administration of these polysaccharides at low, medium, and high doses (50, 100, 200 mg/ [kg·d]) for 60 days effectively reduced the expression of apoptosis-related factors, such as Bcl-2, Bax, and Caspase-3, in the brains of mice with AD-like phenotypes. This observation suggested that the anti-AD mechanism of *Salvia miltiorrhiza* polysaccharides might curtail neuronal apoptosis and exert neuroprotective effects through the inhibition of oxidative stress. Ding *et al*. [78] conducted experiments using cryptotanshinone (4, 20, 40 mg/kg) in a vascular dementia rat model induced by Aβ_1-42_. Their findings revealed that cryptotanshinone exhibited a concentration-dependent effect while suppressing Aβ_1-42_ aggregation. This suppression led to a reduction in Aβ tremor, alleviation of Aβ hydrophobic plaque formation, mitigation of structural and visual alterations in Aβ, and a concentration-dependent suppression of Aβ_1-42_ amyloid protein. Furthermore, cryptotanshinone repressed aggregation in bEnd.3 cells and suppressed the release of inflammatory mediators, such as TNF-α, IL-1β, IL-6, and Caspase-3 activity. These results indicated that cryptotanshinone might exert anti-vascular dementia effects by inhibiting Aβ aggregation in brain vascular endothelial cells.

#### 4.1.2 Chuanxiong Rhizoma (Chuan Xiong)

*Chuanxiong Rhizoma*, derived from the dried rhizome of *Ligusticum chuanxiong* Hort., contains active ingredients, such as phthalides and their dimers, alkaloids, organic acids, polysaccharides, cerebrosides, and sphingolipids. Notably, compounds such as ligustilides, ligustrazine, ferulic acid, caffeic acid, and chlorogenic acid have been emphasized in recent research for their therapeutic properties [79], including anti-inflammatory, antioxidant, and cellular protective properties [80]. Zhang *et al*. (2022) [81] conducted experimental research demonstrating that ligustrazine mitigated brain tissue inflammation in Lipopolysaccharide (LPS)-induced dementia models. This effect was attributed to the reduction of excessive microglial cell activation, resulting in alleviated neuronal damage and improved learning and memory functions. Another study by Zhang *et al*. (2008) [82] on AD model mice found that ligustrazine significantly enhanced learning and memory functions. This enhancement was associated with increased hippocampal ChAT activity, decreased AChE activity, and upregulated M receptor numbers, indicating a beneficial impact on the hippocampal cholinergic system. Furthermore, Liu *et al*. (2014) [83] explored the effects of ligustrazine on AD rat models and uncovered its capacity to attenuate neuronal ROS production and Aβ neurotoxicity by downregulating the receptor for advanced glycation end products (RAGE)-ERK1/2-p38-NF-κB signaling pathway in hippocampal neurons. Consequently, ligustrazine ameliorated brain tissue inflammation in rats with AD-like phenotypes, underscoring its potential as an anti-AD agent with neuroprotective properties.

#### 4.1.3 Gardenia Fructus (Zhi Zi)

*Gardenia Fructus*, derived from the dried fruits of *Gardenia jasminoides* Ellis, has gained attention in modern research for its potential to alleviate AD symptoms. Its therapeutic action in AD primarily involves the modulation of neurotransmitters, anti-inflammatory effects, and the inhibition of neuronal apoptosis. Geniposide is identified as its principal active ingredient. Zhang *et al*. (2020) [84] conducted a network pharmacology study to investigate the anti-AD mechanisms of *Gardenia jasminoides* in the treatment of AD. Molecular docking analysis revealed that over 60% of its active ingredients can bind to targets associated with AD, indicating promising prospects for its use in AD prevention and treatment.

Meanwhile, Zuo *et al*. [85] explored the impact of a 70% ethanol extract of *Gardenia jasminoides* on learning and memory in a Heterogeneity/multi-factors AD (H/MAD) rat model induced by intraperitoneal injection of D-gal, bilateral carotid artery ligation, and intraventricular injection of Aβ. Their results demonstrated that oral administration of 70% ethanol extract (1.05 g/ [kg·d]) effectively improved the learning and memory of rats with AD-like phenotypes. This finding was accompanied by increased CAT activity and reduced AchE content in serum, indicating a role in neurotransmitter modulation. Additionally, Dong Lumeng [86] investigated the anti-AD mechanisms of geniposide using an APP/PS1/Tau protein triple-transgenic AD mouse model. The results revealed that continuous oral administration of geniposide at 100 mg/(kg·d) for 8 weeks significantly reduced the protein levels of glycogen synthase kinase-3β (GSK-3β) in mouse hippocampal neurons and upregulated the levels of Bcl-2. These results suggest that geniposide confers anti-AD effects by reducing Aβ deposition, inhibiting tau protein phosphorylation, and preventing neuronal apoptosis.

#### 4.1.4 Collection of Several Crucial TCMs

Table **3** displays several key TCMs, focusing on their active ingredients and the mechanisms behind their therapeutic effects.

### 4.2 Traditional Chinese Medicine Compound Recipes (TCMCRs)

#### 4.2.1 Buyang Huanwu Decoction (BYHWD)

BYHWD, composed of Astragalus, *Radix Paeoniae Rubra*, *Ligusticum wallichii*, *Angelica sinensis*, *Pheretima aspergillum*, *Persicae Semen*, and *Carthamus tinctorius*, is known for therapeutic effects, such as tonification of qi, promotion of blood circulation, and resolution of stasis to unblock collaterals. The clinical application of BYHWD in the treatment of AD notably enhances cognitive function and functional capacity among AD patients [91]. Fei *et al*. [92] conducted a study using male APP/PS1 double-transgenic mice to clarify the mechanisms of BYHWD in AD-like phenotypes. Their research revealed that continuous intragastric administration of BYHWD at high and medium doses (37.06, 18.53 g/ [kg·d]) for 28 days significantly improved neuronal morphology in the hippocampal CA1 and CA3 regions. The anti-AD effects of BYHWD are likely mediated by several mechanisms, including the preservation of hippocampal neuronal morphology and a reduction in Aβ deposition in mice with AD-like phenotypes, which contribute to its therapeutic properties. Furthermore, these dosages were also observed to effectively suppress the expression of apoptotic factors in the hippocampus, thereby reducing hippocampal neuronal apoptosis and subsequently enhancing learning and memory abilities [93]. Additional research indicated that BYHWD also modulated Aβ metabolism in an AD rat model induced by 24 μmol/L Aβ_25-35_, primarily *via* the RAGE/low-density lipoprotein receptor-related protein 1 (LRP1) pathway [94]. Moreover, BYHWD enhanced its anti-AD properties by improving the permeability of the BBB, which might further facilitate its therapeutic potential [95].

#### 4.2.2 Tongqiaohuoxue Decoction (TQHXD)

TQHXD is composed of Radix Paeoniae Rubra, Ligusticum wallichii, Persicae Semen, Ziziphus jujuba fructus, Carthamus tinctorius, old Allium fistulosum, fresh rhizoma of Zingiber officinale, and Moschus. It is recognized for the pharmacological effect of promoting blood circulation and resolving stasis. The research by Cheng Yanli [96] found that TQHXD enhanced the cognitive abilities and self-care abilities of AD patients by increasing the expression of Brain-derived Neurotrophic Factor (BDNF) and NGF in peripheral blood. The study by Xu Shan [97] administered a combination therapy involving the Western medicine Piracetam and TQHXD with individualized modifications based on patients’ conditions, for the treatment of AD. Their findings indicated that this integrative approach, combining TQHXD with Western medicine, yielded more favorable therapeutic outcomes compared to Western medicine alone. Furthermore, research by Jiang *et al*. [98] demonstrated the capacity of TQHXD to diminish the latency of brainstem-evoked potential in AD patients, increase the amplitude of these potentials, and consequently enhance the cognitive function of AD patients.

#### 4.2.3 Compound Danshen Tablet (CDT)

CDT, comprising TCMs, such as *Salvia miltiorrhiza*, *Panax notoginseng*, and Borneolum, has been studied for its therapeutic potential in AD. Zhang and Hu *et al*. (2018) [99] investigated the therapeutic effects and mechanisms of CDT and donepezil in the treatment of AD. Their study involved serum tests, adverse reaction assessments, and thorough evaluations of patients' psychological status and cognitive abilities. The analyses indicated that CDT, when used in conjunction with donepezil, was more effective in counteracting the toxic effects of EAAs in AD compared to the sole use of donepezil, suggesting a synergistic role in AD prevention and treatment. In the study of Hu *et al*. (2016) [100], APP/PS1 double-transgenic mice were randomly assigned into several groups, including the model group, the CDT group (low, medium, and high doses), and the donepezil group, with age-matched KM mice serving as the normal control. Following two months of treatment, the levels of LRP-1 and RAGE in brain tissue were measured using the Western blot. The results indicated that the model group exhibited appreciably elevated RAGE expression and decreased LRP-1 expression in brain tissue compared to the normal control group.

In contrast, the treatment groups showed a marked reduction in RAGE expression and an increase in LRP-1 expression, with the high-dose CDT group exhibiting the highest LRP-1 expression. Although the donepezil group showed lower expression, the differences between the high-dose CDT and donepezil groups were significant, particularly in LRP-1 expression. The high-dose CDT-treated mice also demonstrated the lowest RAGE expression, although the difference between this group and the donepezil group was not statistically significant. These studies indicate that CDT may potentially treat AD by modulating the expression levels of LRP-1 and RAGE.

#### 4.2.4 Collection of Several Crucial TCMCRs

A selection of several key TCMCRs is presented in Table **4**, detailing their efficacy and mechanisms associated with their therapeutic effects.

## 5 TCMS FOR OPENING ORIFICES, RESOLVING PHLEGM, AND CALMING THE MIND

### 5.1 TCMs and their active ingredients

#### 5.1.1 Poria (Fu Ling)

Poria, derived from the dried sclerotium of *Poria cocos* (Schw.) Wolf, has been extensively investigated for its pharmacological properties. Among its key bioactive components, pachymic acid and *Poria Cocos* Polysaccharide (PCP) have been shown to assume crucial roles in various biological activities, including the elimination of free radicals, the reduction of AChE activity, and the regulation of cellular autophagy [105]. In AD patients, oxidative stress disrupts neuronal stability and contributes to neuronal apoptosis. Pachymic acid exerts its protective effects by inhibiting the ERK/Nrf2 pathway, thereby enhancing the clearance of free radicals and peroxides, ultimately counteracting oxidative stress and neuronal cell death [106].

Furthermore, PCP notably elevates the activity of antioxidant enzymes, such as SOD and CAT, while reducing the levels of MDA [107]. *Poria cocos* has also been shown to significantly reduce AChE activity induced by scopolamine in AD mice, thus enhancing the brain index and demonstrating therapeutic benefits on learning, memory, and sedative-hypnotic abilities. Particularly, PCP significantly improved learning outcomes in AD models [108]. Pachymic acid regulated the expression of autophagy-related proteins, such as LC3-II and Beclin1, through the IGF-1 and PI3K/AKT/mTOR pathways, leading to an enhancement in cognitive function [109]. Additionally, the acidic polysaccharide of *Poria cocos* increased neurotransmitter activities, such as 5-HT, in the hippocampus of rats. Furthermore, it suppressed the NLRP3 inflammasome pathway, reduced the secretion of inflammatory factors in serum, and maintained neurotransmitter balance, thus augmenting neuronal regeneration [110].

#### 5.1.2 Acori Tatarinowii Rhizoma (Shi Chang Pu)

*Acorus Tatarinowii Rhizoma*, the dried roots of *Acorus tatarinowii* Schott, has been extensively researched for its capacity to enhance learning and memory abilities [111, 112]. Its primary chemical constituents include phenylpropanoids, lignans, alkaloids, saccharides, organic acids, and amino acids. Among these constituents, volatile components, such as β-asarone, are considered the principal active ingredient [113]. The experimental research of Wang *et al*. [114] demonstrated that treatment with a combination of *Acorus tatarinowii* and oxiracetam produced superior therapeutic outcomes compared to oxiracetam monotherapy. This synergistic effect might be related to the reduction in the expression of neurotrophic and inflammatory factors in both serum and the hippocampal region of brain tissue. The experimental groups exhibited elevated levels of AD-associated proteins, such as Aβ, tau, p-tau, and inflammatory factors (TNF-α, IL-1β, and IL-6) in serum and the hippocampal tissues. These findings highlight the need for further exploration of the mechanisms underlying the observed therapeutic benefits. Geng *et al*. [115] conducted a study to investigate the impacts of β-asarone, the primary active compound of *Acorus tatarinowii*, on cognitive function and hippocampal neuron apoptosis in rats. The results indicated that β-asarone may improve learning and memory function in a rat model of Aβ-induced dementia by inducing phosphorylation of c-Jun N-terminal kinase (JNK), downregulating the expression of Bcl-2 and Bcl-w, restricting the activation of Caspase-1, and subsequently reducing hippocampal neuron apoptosis. Similarly, research by Shin *et al*. [116] indicated that α-asarone-triggered improvement of memory function might be mediated, in part, by its inhibition of pro-inflammatory cytokines and activation of hippocampal microglial cells. Deng *et al*. [117] demonstrated that *Acorus tatarinowii* volatile oil, in combination with total ginsenosides, might reduce AChE activity, Aβ, and MDA content in the brain cortex and hippocampus of mice induced with D-gal and aluminum chloride. This combination also increased the levels of ChAT and Bcl-2, thereby improving brain tissue damage. Furthermore, Chang *et al*. [118] highlighted the therapeutic mechanisms of active ingredients of *Acorus tatarinowii* in AD, which included antioxidant stress reduction, neuroprotection, anti-apoptotic effects, anti-inflammatory responses, and modulation of central neurotransmitters.

#### 5.1.3 Polygalae Radix (Yuan Zhi)

*Polygalae Radix* is the root of *Polygala tenuifolia* Willd. or *Polygala sibirica* L. It has a longstanding place in TCM, first documented in the ancient Chinese text “Shennong Ben Cao Jing (Shennong's Classic of Materia Medica).” This herb is esteemed for its calming and soothing properties and is traditionally used to treat conditions, such as restlessness, palpitations, insomnia, and forgetfulness. According to the “Shennong Ben Cao Jing,” it is described as: “Beneficial for the nine orifices, enhances wisdom, sharpens the senses, improves memory, strengthens the will, and boosts energy” [119]. The research results of Guo Xinming [120] indicated that tenuigenin could improve the learning abilities and memory of rapidly aging mice. The mechanism underlying this improvement might be associated with a reduction in the levels of AChE in brain tissues. In a study by Ye *et al*. [121], continuous administration of tenuigenin to Aβ-induced AD model rats for 30 days resulted in a significant decrease in the apoptosis rate of hippocampal neurons in the treatment group compared to the model group. This finding was accompanied by an increase in the Bcl-2/Bax expression ratio and a decrease in Caspase-3 expression, suggesting a potential neuroprotective effect.

#### 5.1.4 Collection of Some Crucial TCMs

A collection of some key TCMs is outlined in Table **5**, illustrating their active ingredients and the mechanisms behind their therapeutic effects.

### 5.2 Traditional Chinese Medicine Compound Recipes (TCMCRs)

#### 5.2.1 Kaixin Powder (KXP)

KXP, comprising ingredients, such as *Polygala tenuifolia*, *Panax ginseng*, *Poria cocos*, and *Acorus calamus*, can be traced back to the Tang Dynasty and is documented in Sun Simiao's medical classic “Beiji Qianjin Yaofang.” This TCM formula is primarily used to address memory impairment and forgetfulness, offering therapeutic effects related to nourishing the heart, calming the mind, replenishing qi, and stabilizing determination. Feng *et al*. [126] characterized the pharmacokinetics of six components found in KXP in an Aβ_1-42_-induced AD rat model. The results indicated that α-asarone and β-asarone had relatively short peak times (tmax) and elimination half-lives (t1/2), suggesting rapid absorption and elimination in the AD rat model. Conversely, poricoic acids A and B exhibited rapid absorption but slow elimination. Dehydrotumulosic and dehydrotrametenolic acids had longer tmax and t1/2 values, indicating slow absorption and elimination. Interestingly, dehydrotumulosic acid displayed a biphasic drug-time curve with a longer mean residence time, likely due to pronounced enterohepatic circulation. Huang *et al*. [127] conducted research on the effects of KXP on nitric oxide and AchE in memory-impaired mice. They found that KXP, at doses of 0.3 g/kg and 0.1 g/kg, could repress AchE activity in the mouse brain, with the higher dose group showing a more significant effect than the lower dose group. This finding confirmed that KXP could modulate the cholinergic system to exert anti-AD effects. Lu *et al*. [128] explored the impact of modified KXP on wild-type AD mice. Their observations indicated a reduction in the levels of p-tau protein in the hippocampus of mice in the treatment group. KXP was found to reduce the expression of cyclooxygenase-2, inducible nitric oxide synthase, TNF-α, IL-1β, and IL-6 mRNA in the mice, leading to an improvement in AD symptoms.

#### 5.2.2 Ditan Decoction (DTD)

DTD is composed of nine ingredients, including *Rhizoma Arisaematis*, *Pinellia Rhizome*, *Fructus aurantii immaturus*, *Poria cocos*, tangerine peel, Acorus tatarinowii, *Panax ginseng*, *Caulis Bambusae* in Taeniam, and licorice. Its therapeutic effects involve resolving phlegm and opening orifices, which can alleviate memory impairment in AD patients. Research indicates that in male SD rats of AD models induced by hippocampal injections of Aβ_1-42_, continuous oral administration of DTD at 10 mg/(kg·d) for 4 weeks could activate Phosphodiesterase 2A (PP2A) and inhibit GSK3β expression. This effect, in turn, reduced the excessive phosphorylation of tau protein in AD rats to impart a neuroprotective effect [129]. In research conducted by Peng *et al*. [130], the effects of DTD on D-gal-induced AD rat models were examined. The results demonstrated that continuous oral administration of DTD at low and high doses (4.275, 8.55 g/ [kg·d]) in AD rat models for 4 weeks notably increased GSH-Px levels and antioxidant capacity compared to the untreated model group. This finding suggests that DTD can also achieve its anti-AD effects through the clearance of oxygen-free radicals. Furthermore, other research indicates that DTD renders neuroprotective effects by diminishing the production of brain Aβ in AD model rats [131].

#### 5.2.3 Xixin Decoction (XXD)

XXD is composed of *Panax ginseng*, *Poria cum Radix Pini*, *Pinellia Rhizome*, Citri Reticulatae Pericarpium, *Massa Medicata Fermentata*, licorice, monkshood, *Acorus calamus*, and *Semen Ziziphi Spinosae*. The research by Wang *et al*. [132] suggested that XXD could improve spatial learning and memory abilities in rats with AD-like phenotypes and reduce Aβ_1-42_ deposition in the hippocampal region. The proposed mechanisms include an elevation in neurotrophic factors in the brain-gut axis and the regulation of intestinal microbiota. The study of Gou *et al*. [133] indicated that XXD, its monomeric components, and their combinations could improve learning abilities and memory to varying degrees in AD model rats. The underlying mechanisms may be associated with the activation of the PI3K/Akt signaling pathway, inhibition of neuronal apoptosis, regulation of neuronal autophagy, and suppression of Aβ aggregation within the brain. Due to the complexity of individual components in TCMCRs, further in-depth investigations are still required to elucidate the precise mechanisms of active ingredients in XXD.

Additionally, research by Shao *et al*. [134] demonstrated that XXD-containing cerebrospinal fluid imparted a significant protective effect against Aβ_1-42_-induced cell apoptosis. The mechanisms in this context might involve Aβ_1-42_ leading to an upregulation of ROS accumulation. This increase in ROS could promote the sustained Mitochondrial Permeability Transition Pore (MPTP) opening, which in turn facilitates a reduction in mitochondrial membrane potential. The process is further characterized by an upregulation of Cyt-c expression. These molecular changes subsequently trigger downstream cellular apoptotic cascades, ultimately causing damage to hippocampal neurons in rats.

#### 5.2.4 Collection of Crucial TCMCRs

A selection of some key TCMCRs is presented in Table **6**, displaying their efficacy and the mechanisms of therapeutic effects.

## 6 DISCUSSION

The pathogenesis of AD involves a complex interplay of physiological and pathological factors, such as inflammatory responses, cellular apoptosis, and the disruption of tissue barriers. These complexities present significant challenges to the development of effective treatments. This research focuses on the roles and mechanisms of TCM in alleviating AD and provides an overview of the TCM categories most commonly employed in clinical settings, including nourishing tonic TCMs, blood circulation-promoting and stasis-resolving TCMs, TCMs for opening orifices, resolving phlegm, and calming the mind, as well as TCMCRs. TCMs exert therapeutic effects on AD through various pathways, including the reduction of Aβ accumulation, inhibition of tau protein hyperphosphorylation, modulation of neuroinflammatory responses, counteraction of oxidative stress, regulation of neurotransmitter activity, enhancement of mitochondrial function, regulation of the BBB, modulation of insulin signaling pathways, and alteration of gut microbiota. This study reviews the roles and mechanisms of three key categories of TCMs and their corresponding medicinal compounds to better direct the search for TCM-based treatments for AD. It provides treatment options from the perspective of TCM and offers clarity regarding the principles guiding therapeutic approaches. This review examines the properties and mechanisms of these herbs and seeks to offer insights for subsequent expansion of therapeutic targets for AD. Moreover, it holds the potential to facilitate the development of herbal monomers or extracts that may prove effective in alleviating the symptoms of AD. Despite the promising insights gained from existing research into the anti-aging potential of TCMs, additional clinical trials and further research are needed to validate their efficacy and safety for human application. Future investigations should delve into the mechanisms of herbal remedies in addressing cognitive aging and neurodegenerative diseases. A key focus of this research should involve the identification of active compounds in TCMs and their effects on neural pathways. This knowledge is essential for the successful integration of these compounds into current therapeutic strategies to address aging-related cognitive declines. The modernization of TCMs has driven substantial advancements in the exploration of their potential applications for AD treatment. Nevertheless, this progress encounters several challenges, including unclear dose-response relationships, unstable therapeutic efficacy, and substantial disparities between clinical results and experimental data. To address these challenges and propel further progress in the field, it is crucial to employ modern scientific techniques, encourage interdisciplinary collaboration, and conduct more in-depth research to foster breakthrough innovations in TCM for AD treatment.

## CONCLUSION

The pathogenesis of AD arises from complex interactions between physiological and pathological processes, which poses considerable challenges for effective treatment. The present study focuses on the mechanisms of TCMs in the management of AD and reviews the clinical application of TCMs, which offer a multifaceted approach to targeting AD. This review seeks to identify therapeutic categories, provide guidance for treatment strategies, and clarify the underlying principles of TCM. Moreover, it offers insights for broadening the therapeutic targets for AD and promotes the development of TCM-derived monomers or extracts. While existing studies have recognized the anti-aging potential of TCMs, further clinical trials and research are needed to validate their efficacy and safety. In the future, it is critical to conduct in-depth investigations into the mechanisms of TCMs, particularly focusing on the effects of active compounds on neural pathways. The modernization of TCMs has contributed significant progress and promise to the field of AD therapy. However, challenges remain, including unclear dose-response relationships, variability in therapeutic outcomes, and substantial discrepancies between clinical and experimental findings. To overcome these hurdles, it is imperative to apply modern technologies, foster interdisciplinary cooperation, and pursue more thorough research to facilitate breakthroughs in TCM-based AD treatments.

## Figures and Tables

**Table 1:** Active ingredients and mechanisms of some key traditional Chinese medicines (TCMs).

**TCM**	**Active Ingredients**	**Mechanisms**	**References**
*Glycyrrhiza glabra*	a. Liquiritin b. Glycyrrhizin	a. Exert antioxidative properties. b. Inhibit neurotoxicity induced by Aβ_25-35_ and glutamate. c. Inhibit AChE activity. d. Inhibit tau protein aggregation and prevent Aβ misfolding.	[45-47]
*Polygonatum sibiricum*	*Polygonatum sibiricum* polysaccharides	e. Increase synapse number. f. Alleviate synaptic degeneration. g. Improve synaptic interface structural remodeling.	[48]
*Alpiniae Oxyphyllae* Fructus	*Alpiniae Oxyphyllae* Fructus polysaccharide	h. Regulate neurotransmitters. i. Suppress AChE activity. j. Inhibit tau protein aggregation.	[49]
*Lycium barbarum*	a. *Lycium barbarum* polysaccharide b. quercetin	k. Reduce β-amyloid protein deposition. l. Inhibit tau protein aggregation.	[50]
*Cistanche deserticola*	c. *Cistanche deserticola* polysaccharides d. Total glycosides of *Cistanche deserticola*	m. Maintain normal acetylcholine levels in the brain. n. Enhance antioxidative capabilities.	[51, 52]
*Polygonum multiflorum*	Stilbene glycoside	o. Inhibit c-Jun N-terminal kinase (JNK) phosphorylation. p. Regulate mitochondrial pathway-associated protein activity.	[53]
*Schisandra chinensis*	a. Schisandrone b. Schizandroside c. Deoxyschizandrin	q. Suppress inflammatory factor expression. r. Confer antioxidative properties. s. Reduce tau protein phosphorylation. t. Regulate microglial cell polarization.	[54-56]
*Epimedii Folium*	Icariside	u. Reduce protein expression. v. Inhibit cellular autophagy.	[57]
*Rhodiola crenulata*	Salidroside	w. Protect neuronal synapses. x. Suppress neural inflammation.	[58, 59]

**Table 2:** Composition, efficacy, and mechanisms of some key Traditional Chinese Medicine Compound Recipes (TCMCRs).

**Formula Name**	**Composition**	**Efficacy**	**Mechanisms**	**References**
Shuyu Pill	*Rhizoma dioscoreae*, *Poria cocos*, *Angelica sinensis*, *Colla corii asini*, *Ziziphus jujuba* fructus, *Radix Bupleuri*, *Radix Platycodi*, *Panax ginseng*, licorice, *Paeoniae Radix Alba*, *Massa Medicata Fermentata*, *Semen Armeniacae Amarum*, *Saposhnikoviae Radix*, *Ampelopsis Radix*, *Atractylodes macrocephala*, *Rehmannia glutinosa*, *Ligusticum wallichii*, *Semen sojae Germinatum*, *Ramulus cinnamomi*, *Zingiber officinale Roscoe*, tuberous root of *Ophiopogon japonicus*.	Regulate the spleen and stomach, replenish qi, and harmonize the nutrients.	a. Inhibit the expression of inflammatory factors. b. Regulate neurotransmitters.	[71]
Yizhi Fangdai Formula	Prepared rehmannia root, *Cornus officinalis*, *Alpiniae Oxyphyllae* Fructus, *Cervi Cornus Colla*, *Astragalus membranaceus*, *Acorus tatarinowii*, *Polygala tenuifolia*, *Curcumae Radix*, *Angelica sinensis*, *Ligusticum wallichii*, Rhubarb root	Nourish the kidney, replenish qi, dispel phlegm, remove stasis, open orifices, and unblock collaterals.	Reduce Aβ deposition.	[72]
Guilu Erxian Jiao	*Cornu Cervi Pantotrichum*, *Plastrum testudinis*, *Panax ginseng*, *Lycium barbarum*	Nourish yin, replenish essence, replenish qi, and tonify yang.	Regulate neurotransmitters.	[73]
Huanshaodan	Prepared rehmannia root, *Rhizoma Dioscoreae*, *Radix Achyranthis Bidentatae*, *Lycium barbarum*, *Cornus officinalis*, *Poria cocos*, *Eucommia ulmoides*, *Polygala tenuifolia*, *Morinda officinalis*, *Schisandra chinensis*, *Foeniculi fructus*, *Fructus Broussonetiae*, *Cistanche deserticola*, *Acorus tatarinowii*, *Ziziphus jujuba* fructus	Nourish the essence, replenish the blood, warm and nourish the spleen and kidneys.	Regulate neurotransmitters.	[74]

**Table 3:** Active ingredients and mechanisms of several key Traditional Chinese Medicines (TCMs).

**TCM**	**Active Ingredients**	**Mechanisms**	**References**
*Carthamus tinctorius*	Safflower yellow	Alleviate β-amyloid protein (Aβ) induction through cellular activation and inflammation signaling pathways and reduce Aβ deposition.	[87]
*Ginkgo biloba* leaf	Bilobalide, ginkgolide, and *Ginkgo biloba* leaf extract	Reduce Aβ toxicity and protect neuronal cells.	[88]
*Crataegus pinnatifida*	Lignan	Inhibit intrabody Aβ accumulation.	[89]
*Panax notoginseng*	Notoginsenoside	Provide antioxidative stress protection and regulate intestinal flora.	[90]

**Table 4:** Efficacy and mechanisms of some key traditional Chinese Medicine Compound Recipes (TCMCRs).

**Formula Name**	**Efficacy**	**Mechanisms**	**References**
Huangqi Sanxian decoction	Tonify the kidneys and activate blood circulation.	Regulate cellular immune function.	[101]
Tianqi Yizhi Granules	Raise yang and replenish qi; invigorate blood and unblock the collaterals.	Modulate central neurotransmitters in brain tissue.	[102]
Oral Solution of Tongluo Jiunao	Unblock meridians and harmonizing collaterals; tonify qi and invigorate blood.	Alleviate neuro-pathological damage mediated by RAGE and Aβ.	[103]
Bushen Huatan Quyu Decoction	Tonify the kidneys and dispel phlegm; invigorate blood and dispel stasis.	Increase ADAM10 expression and promote the breakdown of APP protein.	[104]

**Table 5:** Active ingredients and mechanisms of some key Traditional Chinese Medicines (TCMs).

**TCM**	**Active Ingredients**	**Mechanisms**	**References**
*Citri Reticulatae Pericarpium*	Flavonoid, nobiletin	Reduce abnormal accumulation of neurotoxic amyloid β peptide, inhibit excessive phosphorylation of tau protein, and decrease neuronal apoptosis.	[122]
*Liquidambar orientalis*	Benzoic acid	Inhibit the release of inflammatory factors and reduce cellular damage.	[123]
Moschus	Muskone	Reduce Aβ levels and enhance synaptic plasticity.	[124]
Borneolum	Borneolum	Repair damaged neurons	[125]

**Table 6:** Efficacy and mechanisms of some key Traditional Chinese Medicine Compound Recipes (TCMCRs).

**Formula name**	**Efficacy**	**Mechanisms**	**References**
Yuanzhi Powder	Replenish qi, transform phlegm, and open orifices.	Inhibit excessive phosphorylation of tau protein.	[135]
Anshen Dingzhi Formula	Tranquilize spirit, calm mind, transform phlegm, and calm fright.	Suppress neuroinflammatory responses and regulate phosphorylation levels of tau protein.	[136]
Modified Diankuang Mengxing Decoction	Resolve phlegm, invigorate blood, and regulate the flow of qi and blood.	Reduce Aβ deposition.	[137]
Shexiang Baoxin Pill	Diffuse aroma and warm, replenish qi, and strengthen the heart.	Inhibit the formation of Aβ fibrils in Aβ_1-42_-induced PC12 cells.	[138]
Tiaoxin Recipe	Invigorate blood, clear the nutrients, calm the heart, and tranquilize the spirit.	Improve neurotransmitter activity, regulate synaptic plasticity, suppress central inflammatory responses, and alleviate oxidative stress damage.	[139-141]

## LIST OF ABBREVIATIONS

ACh: Acetylcholine
AChE: Acetylcholinesterase
AD: Alzheimer's Disease
APP: Amyloid Precursor Protein
AS-IV: Astragaloside IV
Aβ: Amyloid β-Protein
BACE1: β-Site Amyloid Precursor Protein Cleaving Enzyme 1
BBB: Blood-Brain Barrier
BDNF: Brain-Derived Neurotrophic Factor
CAT: Catalase
ChAT: Choline Acetyltransferase
D-gal: D-galactose
EAAs: Excitatory Amino Acids
EPO: Erythropoietin
FMN: Formononetin
GPs: Ginseng Polysaccharides
GSH-Px: Glutathione Peroxidase
GSK-3β: Glycogen Synthase Kinase-3β
H/MAD: Heterogeneity/Multi-Factors AD
hBMECs: Human Brain Microvascular Endothelial Cells
IBO: Ibotenic Acid
JNK: Jun N-terminal Kinase
LRP1: lipoprotein Receptor Related Protein 1
MDA: Malondialdehyde
MPTP: Mitochondrial Permeability Transition Pore
MWM: Morris Water Maze
NF- κB: Nuclear Factor κB
NFTs: Neurofibrillary Tangles
NGF: Nerve Growth Factor
PCP: Poria Cocos Polysaccharide
PP2A: Phosphodiesterase 2A
PPD: protopanaxdiol
PRR: Processed Rehmanniae Radix
SOD: Superoxide Dismutase
TCM: Traditional Chinese Medicine
ZO-1: Zonula Occludens-1
